# Development and evaluation of an “emergency access button” in Danish out-of-hours primary care: a study protocol of a randomized controlled trial

**DOI:** 10.1186/s12913-017-2308-y

**Published:** 2017-05-31

**Authors:** J. F. Ebert, L. Huibers, F. K. Lippert, B. Christensen, M. B. Christensen

**Affiliations:** 1 Section for General Medical Practice, Department of Public Health, Aarhus University, Bartholins Allé 2, DK-8000 Aarhus C, Denmark; 2 Research Unit for General Practice, Department of Public Health, Aarhus University, Bartholins Allé 2, DK-8000 Aarhus C, Denmark; 3 Emergency Medical Services Copenhagen, The Capital Region of Denmark, Telegrafvej 5, DK-2750 Ballerup, Copenhagen Denmark

**Keywords:** Denmark, After hours, Primary care, Health services, Accessibility, Triage

## Abstract

**Background:**

Out-of-hours (OOH) health care for acute medical problems is often challenged by long waiting time for callers in need of advice and triage. Allowing patients to bypass the OOH telephone waiting line may increase patient satisfaction and provide them with a feeling of safety. We aimed to develop an “emergency access button” enabling patients to bypass the normal telephone waiting line in out-of-hours primary care (OOH-PC) if they perceive their condition to be critical and to evaluate the effect of introducing the button in terms of patient satisfaction and their feeling of safety.

**Methods:**

All patients calling the OOH-PC in two different Danish health care regions during three months will be included in this randomized controlled trial. Data will be collected through two questionnaires developed for this study: a pop-up questionnaire on the relevance of bypassing the normal waiting line to be completed by triage professionals after patient contact and a paper/electronic questionnaire on perceived safety and satisfaction with the emergency access button to be completed by the callers. These questionnaires were developed and validated using external and internal expert feedback, focus group interviews and a two-week field test. The study will be conducted over three months with an estimated user-rate of the emergency access button of 3%.

**Discussion:**

We have developed an emergency access button and we now want to investigate whether this new option will influence upon the level of satisfaction and the feeling of safety in the calling patients. Additionally, the study will reveal the assessed relevance of the decision to bypass the line by triage professionals.

**Trial registration:**

Registered as NCT02572115 at Clinicaltrials.gov on October 5^th^ 2015.

## Background

Many countries offer several access options into the healthcare system for patients with acute health problems [1]. These include out-of-hours primary care (OOH-PC), emergency departments (ED) and emergency medical call centers such as the Emergency Medical Dispatch Center (EMDC-112) receiving emergency calls through the European emergency number 1-1-2. Although these entities target different healthcare needs, their patient population seems to be partly overlapping.

Out-of-hours care is an important part of primary healthcare; it is the point of entrance into the health care system for the many patients who contact health care outside normal working hours, i.e. between 4 p.m. and 8 a.m. on weekdays, in weekends and during holidays [2]. Out-of-hours care is thus intended to provide care for many different patient groups, and the services offered depend on the nature and the severity of the perceived health problem. The individual patient’s decision to contact a specific setting implies that the patient selects and chooses the point of access to out-of-hours care, and this choice also influences the care pathway [3]. An “inappropriate” choice may result in serious delay of treatment or insufficient intensive care, for example if primary care is contacted instead of the EMDC-112 in life-threatening situations [4]. However, overuse or overtreatment is a potential risk if patients call the EMDC-112 for minor problems [5, 6].

OOH-PC is provided by general practitioners (GPs) in large-scale organizations in four out of five Danish regions [2], whereas one region offers a medical helpline (MH-1813) serving as a publicly run call center. All persons calling OOH-PC or the MH-1813 must wait in line, even if the health problem is perceived as highly urgent or even life-threatening. As there is no option to bypass the telephone waiting line, the only alternative to waiting is calling the EMDC-112, which is intended for life-threatening situations that require immediate medical response, for instance dispatch of an ambulance. A previous study showed that approximately 1% of all OOH-PC telephone contacts are triaged directly to the EMDC-112. Additionally, approximately 5% of all patients estimate their condition as potentially life-threatening when calling OOH-PC [7]. One of the main reasons for calling OOH-PC is worry [8–10], and callers may experience distress due to long waiting time when calling with a perceived acute health problem [11]. A combination of long waiting time, worry and distress might lead to a low feeling of safety in callers.

Introducing an option to bypass the telephone waiting line could prompt a feeling of safety in callers and thus reduce the level of distress in medical situations. The Danish bypass option is inspired by a similar option in the Netherlands, but no research is currently available on the subject. The bypass option may increase the callers’ satisfaction with OOH-PC and the MH-1813 in general [12, 13]. It is important to ensure that such option is not misused as this might cause unnecessary long waiting time for the callers who choose not to bypass the line. Thus, we aim to develop and evaluate an emergency button that enables the caller to bypass the telephone waiting line in OOH-PC in the Central Denmark Region and in MH-1813 in the Capital Region of Denmark. We will study the frequency of emergency button use, the characteristics of the callers who choose to use the button compared with non-users and the effect of the bypass option on the callers’ reported feeling of safety and their level of satisfaction. Furthermore, we aim to investigate the triage professionals’ evaluation of the relevance of using the emergency button.

## Methods

### Design and setting

We will conduct a parallel randomized controlled superiority trial at the OOH-PC in the Central Denmark Region and in MH-1813 in the Capital Region of Denmark. These two settings were selected because they represent the two main organizational models for out-of-hours acute care in Denmark; they differ in access method, triage professional profile and waiting time (see Table 1). The comparison of data on emergency button use between these two regions provides us with documentation of the performance of the intervention and its generalizability. The study was developed in accordance with the SPIRIT guidelines [14].

**Table 1:** Differences in out-of-hours telephone setup

Subject	OOH-PC in the Central Denmark Region	MH-1813 in the Capital Region of Denmark
Triage professionals	General practitioners^e^	Nurses and doctors^f^
Waiting time (90^th^ percentile, 2015)	7 min.^c^	16 min. 35 sec^d^
Waiting time (mean, 2015)	2 min. 30 sec^c^	6 min. 51 sec^d^
Waiting time (median, 2015)	1 min. 8 sec^c^	4 min. 29 sec^d^
Contacts per year (2014)	697,000^a^	911,000^b^
Inhabitants (per 1 Jan. 2015)	1,282,750^a^	1,760,000^a^
Contacts per inhabitant	0.54	0.52

Sources: ^a^Statistics Denmark, ^b^Capital Region of Denmark, ^c^Central Denmark Region, technical dep., ^d^Emergency Medical Services Copenhagen, Capital Region of Denmark, ^e^or doctors in final phase of GP specialist training, ^f^Can be GPs, doctors with other specialties than GP or doctors in training

In the Capital Region of Denmark, patients must call the MH-1813 if they need urgent medical advice; the telephone is primarily answered by triage nurses. They have the option to give telephone advice, forward the call to a doctor (most often with other specialties than general practice), triage to a consultation at an ED, refer to hospitalization, plan a home visit or dispatch an ambulance or forward the call to the EMDC-112. In the Central Region of Denmark, OOH-PC is run by GPs; all telephone calls are answered and triaged by GPs. They can offer a telephone consultation, triage to a clinic consultation or a home visit, refer directly to an ED/hospitalization or forward the call to the EMDC-112. Both the GPs in OOH-PC and the nurses/doctors in MH-1813 are referred to as *triage professionals*.

### Participants

All callers contacting the OOH-PC in the Central Denmark Region and the MH-1813 in the Capital Region of Denmark in the study period are invited to participate. A welcome message on the telephone will inform about the study and give the caller the opportunity to decline participation by pressing “1”. We will not include patients who have died since the index call or patients in the age group 14–17 years because of confidentiality issues (they sometimes contact OOH-PC without the knowledge of their parents). Callers can be included only once, even if they have several contacts during the study period.

### Intervention

**Fig. 1:**
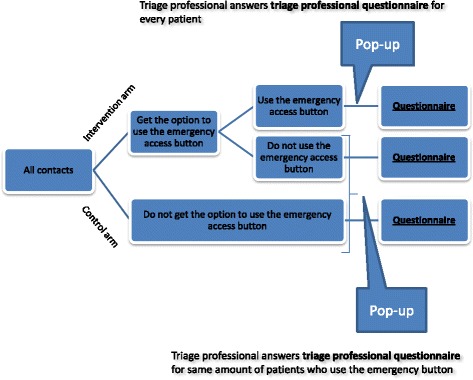
Flowchart of intervention

Callers are routinely asked to type in the unique civil registration number (CRN) of the patient whom the call concerns on their telephone when calling OOH-PC and MH-1813; approximately 90% of all callers provide this information. The callers will be allocated into two arms according to their date of birth (even versus uneven date of the month), which is part of the CRN. This will ensure that a patient will be randomized to the same arm if s/he calls multiple times during the study period. The included patients will receive only one questionnaire regarding their first contact. One arm will be the control group, whereas the other arm will be the intervention group (see Fig. 1).

For those in the intervention arm, the message on the answering machine will inform callers of the option to bypass the waiting line by pressing “9”. If the caller chooses to press “9”, the call will be answered by the next available triage professional. The triage professional will not know whether the caller has used the emergency button or not when answering the call. Callers in the control arm will get the usual message on the answering machine without the option to bypass the line. If the caller does not type in the CRN, s/he will not be included in the study and will be redirected to receive the usual message on the answering machine.

### Data collection

Data for this study will be collected by two types of questionnaires and information from the electronic patient record system.

### Triage professional questionnaire

A questionnaire will pop up on the work station of the triage personnel for each emergency button user immediately after termination of the contact. For every pop-up questionnaire concerning an emergency button user, a similar questionnaire will pop up for a random group of non-users in the intervention arm and for a random group of callers in the control arm (see Fig. 1).

The pop-up questionnaire contains seven items and can be completed in less than one minute. Three validated items origin from a GP pop-up questionnaire used in an earlier study on OOH-PC contacts [15]. These questions focused on reason for encounter, level of urgency and probable diagnosis. Four additional items were developed specifically for the new pop-up questionnaire; two measuring the relevance of the caller’s choice to bypass the line as seen from a strictly medical point of view and a psychosocial point of view and two focusing on the caller and the reason for encounter. The face validity was assessed with the help from seven GPs with solid OOH-PC experience. The GPs provided feedback on the formulation of questions and on the feasibility of answering the questionnaire when being on duty. The content validity was assessed through ten internal feedback rounds within the author group and with the research department’s communication specialist. The questionnaire was field-tested during two weeks of January 2016 (see Table 2).

**Table 2:** Experiences from the field test

Call statistics	Triage professional questionnaire	Caller questionnaire
3% bypassed the line	Easy to complete	43% response rate
38% declined participation	Approx. 70% GP participation from start of shift	Focus groups: - Items easy to understand - Rephrase three items - Support of the intervention
10% did not type in civil registration number		

### Caller questionnaire

Questionnaires will be sent to all callers for whom the triage professional has completed a pop-up questionnaire. The questionnaire is intended to be answered by the caller, who will also be the patient in most cases. As the questionnaire is posted to the patient for whom the CRN was entered, it will not always be possible to get the caller to answer the questionnaire, for example if the caller was not the patient or a relative in which case the patient will be asked to complete the questionnaire. The caller questionnaire contains 23 questions and differs for the three groups on only one topic, depending on the use of the emergency button (see Table 3). The developed questionnaire consists of a mix of validated items from former studies [7], items from existing validated scales, i.e. SF-36 [16] and GAD-2 [17], and newly developed items. Content validity was assessed through ten internal feedback rounds within the author group and with the research department’s communication specialist. Face validity was assessed through a field test, which was conducted during two weeks of January 2016, and through two semi-structured focus group interviews, which were performed in May 2016; these interviews focused on the definition and understanding of safety from a caller perspective and served to further clarify and specify the wording of the items (see Table 3).

**Table 3:** Participant groups and questionnaire content

Groups	Triage professional questionnaire	Caller questionnaire
All three groups: common content of questionnaire	Reason for encounter - Symptom - Possible diagnosis Severity of condition New illness/injury or exacerbation of chronic illness/injury	Background information: - Gender, age, civil status, educational level, ethnicity - Mental and physical health Questions regarding the specific contact: - Reason for encounter - Severity of condition - Caller’s expectations - Satisfaction with the specific contact General questions: - General feeling of safety with the service - General satisfaction with different health services
	Additional questions	Additional questions
Intervention group: “users” Get the option to bypass the line	Questions regarding the evaluation of the relevance of bypassing seen from a medical perspective Questions regarding the evaluation of the relevance of bypassing seen from a psychosocial perspective	Questions regarding the emergency access button: - Feeling of safety - Recommendation of implementation
Intervention group: “non-users” Get the option to bypass the line, but choose not to	Question regarding whether the caller should have bypassed the line or not	Questions regarding the emergency access button: - Feeling of safety - Recommendation of implementation
Control group	Question about whether the caller should have bypassed the line or not	

The caller questionnaire will be sent a few days after the contact to reduce the risk of recall bias. We plan to use the digital mailbox linked with the unique Danish CRN (this mailbox is currently being tested for reliability and response rate) and surface mail for patients without a digital mailbox (approximately 10%). The digital mailbox approach will allow us to email a unique link for an internet questionnaire, which is easily accessible for callers. If the patient is less than 14 years old, the questionnaire will be addressed to the parents. For electronic questionnaires, a reminder will be sent one week and two weeks after the contact. For paper questionnaires, one reminder will be sent three weeks after the contact (for cost reduction).

### Electronic patient record system

We collect information for all contacts in the study period from the electronic patient record systems of both OOH-PC and the MH-1813: CRN of the patient, date and time of contact, triage outcome (i.e. telephone consultation, clinic consultation, home visit, referral to ED or referral to hospitalization), use of emergency button and estimated waiting time in the telephone line at the time of the call. If the caller has chosen to bypass the line, we will also collect information about the length of the waiting time at the time of the bypass.

## Outcome measures

### Primary outcome measures

1. Frequency of bypassing among callers
2. Caller satisfaction and feeling of safety; button users versus non-users
3. Relevance of button usage assessed by triage professional

### Secondary outcome measures

1. Characteristics of button users versus non-users (e.g. age, gender and reason for encounter)
2. Reasons for using/not using the emergency access button

## Study period and power calculations

We performed power calculations to assess the number of respondents needed to answer our main research question on feeling of safety and to assess the expected duration of the study period to ensure valid estimates of emergency button use. As both the number of button users and the response rate of caller questionnaires can influence the length of the study period, we performed two power calculations: one based on response rate and another based on number of button users.

### Response rate

Our main outcome measures on satisfaction and feeling of safety will both be measured by a 5-point Likert scale. We want to be able to detect a mean difference of at least 0.3 between button users and non-users. If we assume that the sample standard deviation is 1, the significance level is 5%, the power is 95% and given a mean of 3, we need a total of 290 completed patient questionnaires for each of the three groups to be able to detect a difference of 0.3 between two groups.

**Fig. 2:**
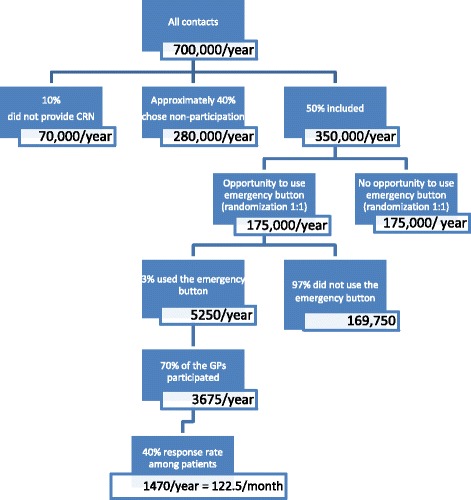
Flowchart of power calculation

Our field test showed that approximately 40% of all callers chose not to participate in the study, and 10% did not type in their CRN, thus leaving them out of the randomization (Fig. 2). In 2014, approximately 700,000 contacts were made to the OOH-PC in the Central Denmark Region [18]. This gives us a study population of 50% out of 700,000, i.e. 350,000 for one year. Of these, 50% would be randomized into the intervention arm (175,000); 3% of these would use the emergency button (5,250). The field test also showed that approximately 70% of the triage personnel chose to participate in the study (3,675) and that the response rate of the caller questionnaire was approximately 40% (1,470). Thus, we will get 123 answered questionnaires from button users per month if the above-mentioned conditions are taken into account. To collect 290 questionnaires per group, the duration of the study period must be approximately 2.4 months.

### Number of button users

To investigate the frequency of bypassing the line, we need to determine the user rate. Our field test in the OOH-PC service in the Central Denmark Region showed a user rate of approximately 3% for the emergency button, which gives 5,250 users in one year (see Fig. 2). To ensure satisfactory power in our calculations regarding choice of the bypass option, we aim for a 95% confidence interval (CI) of +/− 0.8% (2.2%; 3.8%). This means that we need at least 1,950 button users, which will require a study period of 2.7 months.

On the basis of the calculations from the field test of the OOH-PC services in the Central Denmark Region, we expect that a period of no more than three months will be required to complete the study. As more contacts per year are directed to the MH-1813 in the Capital Region of Denmark (approximately 910,000) and the response rate of the MH-1813 triage professionals is also expected to be higher due to an obligation by the management to participate in the study, the study period is expected to run for less than three months. The study is planned to take place at the same period of time in the two selected settings.

## Analyses

We will provide a descriptive analysis of three groups: callers who chose to bypass the waiting line, callers who chose not to bypass the line and callers from the control group. In addition, we will perform univariate analyses comparing the callers in the three groups (e.g. caller characteristics, reason for encounter and motives for calling). Descriptive analyses will be performed using Student’s *t*-test for data following a normal distribution, Mann–Whitney *U*-test for non-normally distributed data and chi-square test for categorical data. For our primary outcome measures, i.e. patient satisfaction and feeling of safety, we will perform intention-to-treat analyses and subgroup analyses. Associations between relevant use of the emergency access button, level of urgency (as assessed by triage professional) and caller characteristics will be explored by multivariate binomial regression taking clustering at triage professional level into account. Associations between callers choosing to bypass the line, satisfaction with having the option and the reasons for using the option will be assessed using multivariate binomial regression models.

## Discussion

A strength of this study is the valid and easy-to-use method of collecting data from triage professionals, i.e. a pop-up questionnaire on their work station [15]. This method lowers the risk of recall bias in the triage professionals because the pop-up questionnaire is completed immediately after the patient contact. Additionally, the large patient flow into the OOH-PC/MH-1813 services in the two regions enables us to include a considerable number of callers in a relatively short time period. Furthermore, the use of an intervention in two organizations with differences in terms of access, triage professional profile and waiting time, and yet similar patient populations, will allow us to explore the use of the intervention in detail. The use of a robust intervention makes it possible to later implement the intervention into other similar OOH-PC settings relatively easily. Also, we have performed a field test of the emergency access button and the questionnaires and have also conducted focus group interviews, which showed that both questionnaires were easy to complete. The obtained information also gave us an idea of the user rate (3%); this is in line with former experiences in the Netherlands, which suggest that overuse is not a serious risk.

However, the fairly low participation rate from both triage professionals and callers constitutes a limitation of this study. We found that 38% of all callers declined to participate and another 10% did not type in their CRN, which is a potential source of selection bias. The level of assessed urgency might be higher among these callers, and they may feel that they are not in a state to participate in surveys. The response rate for the caller questionnaire was approximately 40% in the field test. Even though this is comparable to similar studies [19], this could induce further selection bias. We plan to distribute the caller questionnaire by digital post, which is expected to give a higher response rate. Furthermore, the data collection with use of reminders could introduce recall bias. A reminder will be sent one and two weeks after the contact; the reasons for using the button might be difficult to retrieve at this time point. To reduce this risk, we have predefined categories to help the caller.

### Perspectives

Firstly, this study will provide knowledge on the feasibility and the effects of implementing an option to bypass the telephone waiting line in OOH-PC and the MH-1813. A well-functioning option to bypass the telephone waiting line in case of emergency may improve the access of acute out-of-hours services in Denmark as well as limit non-relevant use of the EMDC-112.

Secondly, even though the percentage of callers who may benefit from bypassing the waiting line is expected to be relatively limited, the total number of contacts to OOH-PC and the MH-1813 is extensive, which means that the absolute number of patients who actually benefit from this simple intervention is likely to be substantial. As the waiting time in the OOH-PC telephone services is a known source of frustration to most people [11], an option to bypass the waiting line for acute matters could potentially provide greater satisfaction and feeling of safety in many people.

Moreover, it will be studied whether callers will manage to use such a bypass option as intended. On the basis of the field study, our estimates suggest that 3% will use the emergency button; this indicates that the option of bypassing the line will not be misused. Together with an assessment of the relevance of the choice of bypassing, this information can be used to decide whether the intervention should be implemented nationwide.

## Abbreviations

CRN: Civil registration number (social security number)
ED: Emergency department
EMDC-112: Emergency medical dispatch center 112 (emergency number for life-threatening injury or illness and request of emergency ambulances)
GP: General practitioner
MH-1813: Medical helpline 1813 (medical call center operated by nurses and doctors with either no medical specialty or other specialty than general practice)
OOH-PC: Out-of-hours primary care.
